# Association between high school students’ cigarette smoking, asthma and related beliefs: a population-based study

**DOI:** 10.1186/s12889-016-3579-7

**Published:** 2016-09-01

**Authors:** Resa M. Jones, Kara P. Wiseman, Marina Kharitonova

**Affiliations:** 1 Division of Epidemiology, Department of Family Medicine and Population Health, School of Medicine, Virginia Commonwealth University, 830 E. Main St., 8th Floor, Box 980212, 23298-0212 Richmond, VA USA; 2 Massey Cancer Center, Virginia Commonwealth University, Richmond, VA USA; 3 Center on Health Disparities, Virginia Commonwealth University, Richmond, VA USA

**Keywords:** Adolescents, Asthma, Smoking, Smoking-related beliefs

## Abstract

**Background:**

Smoking has a detrimental effect on the symptoms and severity of asthma, a common chronic disease among adolescents. The purpose of this study was to examine the association between asthma and smoking among high school students and assess provider-patient communication with asthmatic adolescents regarding smoking and adolescents’ beliefs about the harms of smoking.

**Methods:**

In fall 2014, data from high school students, ages 14–18 years, completing the 2009-2010 Virginia Youth Tobacco Survey (*N* = 1796) were used in descriptive analyses and multivariable logistic regression models adjusting for model-specific confounders as appropriate.

**Results:**

Overall, an estimated 19 % of high school students in Virginia smoked and 16 % had asthma. Odds of smoking did not differ by asthma status; however, asthmatics had 1.5 times higher odds of being asked if they smoke (95 % CI 1.06–2.13) and being advised not to smoke by a health professional (95 % CI 1.10–2.14) compared to non-asthmatics. Asthmatics who believed second-hand smoke or smoking 1–5 cigarettes/day was not harmful had respectively 4.2 and 2.8 times higher odds of smoking than those who thought each was harmful. Further, asthmatics who thought smoking 1−2 years is safe had 3.4 times higher odds of smoking than those who did not (95 % CI 1.57–10.1).

**Conclusions:**

While asthmatic adolescents are just as likely to smoke as non-asthmatics, less healthy beliefs about the risks of smoking increase the odds of smoking among asthmatics. Thus, targeted asthma-specific smoking prevention and education to change attitudes and beliefs could be an effective tool for adolescents.

## Background

Asthma is one of the most common chronic diseases among children and youth in the United States [1]. Approximately 14 % of children aged 17 and under have ever been diagnosed with asthma [2] Among high school students, primarily ages 14–18 years, about 11 % currently have asthma [3]. Asthma affects everyday activities, such as playing, exercise, school attendance, and sleeping, as well as emotional health; [1, 4] however, asthma is possible to manage and control with medication and behavior modification [1].

All asthma management guidelines state that patients should be strongly advised not to smoke [5]. Adolescents with asthma are expected to avoid smoking because it might initiate or aggravate asthmatic symptoms [6]. Further, asthmatics who smoke have more asthma attacks, worse pulmonary function and higher rates of hospitalization [7]. In addition, smoking may reduce the effectiveness of daily control medicine [8]. Overall, about 20 % of U.S. high school students [3] and Virginia high school students [9] smoke, which compromises their current and future health. While one study found that asthma in childhood reduces smoking initiation in male adolescents [10], the majority of previous studies assessing smoking among asthmatic adolescents show that they are more likely to smoke than non-asthmatic adolescents [11–15]. In addition, a cohort study of adolescents examined smoking and asthma and found a bidirectional relationship [15]. Some studies have found that while the demographic and behavioral predictors of smoking could be the same for adolescents with and without asthma [11], some differences in motivation exist [16, 17]. Other research suggests that asthmatic adolescents may make a more active decision to start smoking than adolescents without asthma [17].

Little is known about how provider communication might impact adolescents’ smoking or what influences smoking behavior among asthmatics. Thus, to better inform intervention development and identify areas for education, the objectives of this study are to: (1) describe the prevalence of those with and without diagnosed asthma among Virginia high school students and the sub-group who smoked in the past 30 days, (2) examine the association between the prevalence of asthma and smoking, (3) evaluate provider-patient communication regarding smoking and examine adolescents’ beliefs about the harms of smoking by asthma status, and (4) examine the association between smoking and provider-patient communication regarding smoking and adolescents’ beliefs about the harms of smoking.

## Methods

### Study sample

A two-stage cluster sample design was used to produce a representative sample of Virginia high school students (grades 9–12; majority are ages 14–18 years) for the Virginia Youth Tobacco Survey (Virginia YTS) [9]. Stage one of the sampling included all Virginia public schools containing grades 9, 10, 11, or 12. Schools were selected with probability proportional to school enrollment size. Stage two consisted of systematic equal probability sampling (with a random start) of classes from each participating school. All second period classes in the selected schools were included in the sampling frame. All students in these selected classes were eligible to participate in the survey.

Seventy-two percent of randomly selected high schools agreed to participate (36 of 50) and the student response rate (completed questionnaires divided by total number of students) for students in the selected class periods in participating high schools was 81.9 % (*N* = 1827 of 2232) [9]. The overall response rate was 58.9 % [9]. This study includes respondents ages ≥14 years who answered the primary questions of interest (*N* = 1796; *n* = 16 students <14 years were excluded). This study was approved by the Virginia Commonwealth University Institutional Review Board.

### Survey

The Virginia YTS is a self-administered, written questionnaire based on the validated Youth Tobacco Survey and Youth Risk Behavior Survey designed by the Centers for Disease Control and Prevention (CDC) [3, 9]. The survey, distributed in classrooms during the 2009 – 2010 academic year, included questions about tobacco use, tobacco-related psychosocial constructs, asthma, health care, and demographics.

### Measures

Students were asked “During the past 30 days, on how many days did you smoke cigarettes?” Responses were combined to create a dichotomous variable indicating whether or not the student had smoked in the past 30 days. Students were also asked whether “a doctor or nurse ever told [them] that [they] have asthma?” Responses were coded to reflect three categories-Yes, and still have it now; Yes, but don’t have it anymore; and No.

Several questions measured smoking-related variables. Students provided information on whether “During the past 12 months, did any doctor, dentist, nurse, or other health professional ask you if you smoke?” and “During the past 12 months, did any doctor, dentist, nurse, or other health professional advise you not to smoke?” (“yes”, “no”, and “don’t know/not sure” were dichotomized as “yes” or “no”, which included “don’t know/not sure”). Students also answered knowledge/belief questions relating to the harms of smoking, with response options of “definitely yes”, “probably yes”, “probably not”, and “definitely not”, which were recoded as binary variables to reflect “yes” or “no” responses (i.e., “Do you think young people risk harming themselves if they smoke from 1 to 5 cigarettes per day?” “Do you think it is safe to smoke for only a year or two, as long as you quit after that?” and “Do you think the smoke from other people’s cigarettes is harmful to you?”) Students also provided information on gender and age as well as race (i.e., White, Black, and Other), whether they lived with a smoker, the disposable funds they had to spend in any way in the last 4 weeks (coded as none, $1–$10, $11–$20, $21–$50, and > $50), how many of their four closest friends smoke (coded as ≥1 or none), and rules about smoking in the home or car (coded as always/sometimes allowed or never allowed).

### Statistical analysis

SAS 9.4 PROC SURVEYFREQ, PROC SURVEYREG, and PROC SURVEYLOGISTIC were used for data analysis with appropriate weights and strata given the sampling scheme of the Virginia YTS. Descriptive statistics were calculated for demographics of the overall sample and among those who smoked in the last 30 days and to determine the prevalence of asthma and smoking. Differences in smoking-related communication with health professionals and beliefs about the harms of smoking by asthma status were assessed. Logistic regression was used to assess both the association between asthma and smoking (dependent variable) among all students and the relationship between health professional communication about smoking and individual beliefs about the harms of smoking and smoking behavior (dependent variable) among asthmatics. We determined whether there was evidence of effect modification for age, race, or gender for all models. Possible confounders (i.e., student’s gender, age, race, whether they lived with a smoker, amount of disposable funds available to spend in any way in the last 4 weeks, friends’ smoking, rules about smoking in the home, and rules about smoking in the car) were assessed for the regression models using the 10 % change-in-estimate rule as conceptually appropriate [18]. The final multivariable logistic regression models provide odds ratios (OR) and confidence intervals (CI) adjusted for model-specific confounders.

## Results

Table 1 provides the estimated demographic characteristics of Virginia high school students, ages ≥14 years. About half were female students and 36.4 % indicated that they live with someone who smokes. Approximately half (55.5 %) were white students and 26.8 % were Black students. Overall, 18.1 % of female and 20.5 % of male students had smoked one or more cigarettes in the past 30 days at the time of the survey. In addition, 16.2 % of the overall sample reported currently having asthma whereas 17.9 % of youth who smoked in the past 30 days reported currently having asthma.

**Table 1:** Descriptive characteristics of overall sample and subgroup of youth who smoked in past 30 days: Virginia high-school students (*N* = 1796)^a^

	Overall sample Weighted frequency (*N* = 216105)	Youth who smoked in past 30 days Weighted frequency (*n* = 39938)
Characteristic	N^b^ (Weighted percent)	Weighted percent
Gender		
Female	105297 (48.9)	18.1
Male	109871 (51.1)	20.5
Race		
Black	56809 (26.8)	14.3
White	117570 (55.5)	19.7
Other	37308 (17.7)	24.1
Lives with smoker		
Yes	72554 (36.4)	26.6
No	126559 (63.6)	13.2
Age		
14	31690 (14.7)	9.0
15	57208 (26.5)	11.5
16	55732 (25.8)	21.8
17	51913 (24.0)	25.6
18 and over	19561 (9.0)	35.1
Asthma		
Yes, currently	31521 (16.2)	17.9
Yes, but not anymore	23241 (12.0)	20.0
Never	139281 (71.8)	18.5

Weighted frequencies and weighted percentages came from descriptive statistics that were calculated taking into account the sampling scheme

^a^Unweighted frequency

^b^Weighted frequency

No evidence of effect modification was found for age, race, or gender. Taking into account asthma status, students who lived with a smoker had 2.57 times higher odds of smoking (95 % CI 1.90–3.48) compared to those not living with a smoker (data not shown in Tables). The odds of smoking among students with current asthma or a previous diagnosis were not significantly different than those who were never diagnosed with asthma after adjusting for the confounder of living with a smoker.

In the past 12 months, a greater percentage of students with current asthma were asked by a health professional about whether they smoked (43.6 %, *p*-value = 0.049) and advised to not smoke (35.4 %, *p*-value = 0.087) compared to those without asthma (Table 2; unadjusted analyses as no evidence of confounding). In fact, after adjustment for model-specific confounders, students with asthma had 1.5 times higher odds of being asked by a health professional whether they smoked (95 % CI 1.06–2.13) and 1.5 times higher odds of being advised to not smoke (95 % CI 1.10–2.14) compared to those who had never had asthma (data not shown in Tables.) Students’ beliefs about the harms of smoking did not significantly vary by asthma status.

**Table 2:** Differences in provider-related communication and smoking beliefs by asthma status: Virginia high-school students (*N* = 1796)^a^

Question	Asthma status			*p*-value^b^
	Yes, currently	Yes, but not anymore	Never	
	Weighted frequency (*n* = 31521)	Weighted frequency (*n* = 23421)	Weighted frequency (*n* = 139281)	
	Weighted population mean	Weighted population mean	Weighted population mean	
During the past 12 months, a doctor, dentist, nurse, or other health professional asked me if I smoke (*n* = 41852)^c^	43.6	36.7	33.6	**0.049**
During the past 12 months, a doctor, dentist, nurse, or other health professional advised me not to smoke (*n* = 32722)^c^	35.4	28.8	26.7	0.087
I think the smoke from other people’s cigarettes is harmful to me (*n* = 130190)^c^	94.3	93.8	94.1	0.983
I think young people risk harming themselves if they smoke from 1 to 5 cigarettes per day (*n* = 128104)^c^	89.9	93.6	92.5	0.457
I think it’s safe to smoke for only a year or two, as long as I quit after that (*n* = 12789)^c^	13.6	13.8	9.2	0.086

*p*-values < 0.05 are shown in bold

^a^Unweighted sample size

^b^ *p*-value of each model assessing differences in population mean prevalence by asthma status among students. All analyses are unadjusted as the following were not confounders: gender, age, race, whether they lived with a smoker, amount of disposable funds available to spend in any way in the last 4 weeks, friends’ smoking, rules about smoking in home, and rules about smoking in car

^c^Weighted sample size of those who gave an affirmative “yes” response to question

Table 3 provides estimates of the association between smoking status and health provider communication and students’ beliefs about smoking for asthmatic students (adjusted for model-specific confounders detailed in Table footnotes). Among students with asthma, smoking status was associated with health professionals asking students about their smoking. Specifically, asthmatics who were asked if they smoked had 2.46 higher odds of smoking than those who were not asked if they smoked (95 % CI: 1.04–5.81). Asthmatic students who thought it was safe to smoke for only 1–2 years had 3.97 times higher odds of smoking than those who did not believe it was safe (95 % CI 1.57–10.06).

**Table 3:** Associations between provider-related communication, smoking beliefs and smoking: Virginia asthmatic high-school students (*n* = 255)^a^

Question		Smoked in past 30 days	Adjusted OR (95 % CI)
		Weighted percent	
During the past 12 months, did any doctor, dentist, nurse, or other health professional ask you if you smoke? (*n* = 11290)^b^	Yes	24.1	**2.46 (1.04, 5.81)** ^c^
	No	17.5	1.0 (referent)
During the past 12 months, did any doctor, dentist, nurse, or other health professional advise you not to smoke? (*n* = 9320)^b^	Yes	24.1	1.77 (0.78, 4.06)^d^
	No	18.2	1.0 (referent)
Do you think the smoke from other people’s cigarettes is harmful to you? (*n* = 29233)^b^	Yes	17.8	1.0 (referent)
	No	31.8	4.17 (0.70, 24.7)^e^
Do you think young people risk harming themselves if they smoke from 1 to 5 cigarettes per day? (*n* = 27925)^b^	Yes	17.4	1.0 (referent)
	No	30.8	2.78 (0.77, 10.1)^f^
Do you think it’s safe to smoke for only a year or two, as long as you quit after that? (*n* = 4238)^b^	Yes	41.7	**3.97 (1.57, 10.1)** ^g^
	No	16.0	1.0 (referent)

95 % confidence intervals that do not include the value 1 are shown in bold

*95 % CI* 95 % confidence interval, *OR* odds ratio

^a^Unweighted sample size

^b^Weighted frequency

^c^Adjusted for race

^d^No significant confounders; crude model

^e^Adjusted for age, race, friends’ smoking, rules about smoking in the car, and disposable spending money

^f^Adjusted for friends’ smoking and disposable spending money

^g^Adjusted for gender and rules about smoking in the home

## Discussion

This study assessed the association between asthma and smoking status as well as high school students’ reported communication about smoking risks with their clinicians, and the association between clinician advice and students’ beliefs about smoking harms and cigarette smoking among asthmatic high school students in Virginia. While several studies have shown that adolescents with asthma are more likely to smoke than those without asthma [11–15], we found asthmatic students were just as likely to smoke as their non-asthmatic peers. The odds of provider communication about smoking and the potential harms of smoking were higher for asthmatic high school students compared to non-asthmatic students. Less healthy beliefs about the actual risks of smoking increase the odds of smoking among asthmatics.

### Smoking prevalence and beliefs about harms of smoking

Despite asthma management guidelines and negative effects of smoking on asthma symptoms, self-reported asthmatic high school students in Virginia are just as likely to smoke as those without asthma. This finding is divergent from the majority of studies that have reported asthmatic adolescents are more apt to smoke than non-asthmatic adolescents [11–15]. However, one previous study found asthma in childhood reduces smoking initiation in male adolescents [10]. Further, a longitudinal cohort study of Dutch adolescents examined smoking and asthma and found a bidirectional relationship. Specifically, at 1-year follow-up, those with self-reported asthma were 1.91 times more likely to begin smoking than those without asthma and those who smoked were 2.86 times more likely to develop symptoms of asthma over the course of the study [15]. These results suggest that adolescents with asthma are more willing to smoke regardless of the unpleasant experiences associated with smoking initiation and the consequences that smoking can induce. Also, differences in motivation exist by asthma status [16, 17]. For example, adolescents with asthma are 1.67 times more likely to report smoking initiation due to peer pressure and a greater proportion of asthmatics use smoking as a means of weight control compared to adolescents without asthma (18.8 % vs 13.1 %) [16]. Evidence also exists that asthmatic adolescents may make a more active decision to start smoking than adolescents without asthma [17].

The vast majority of respondents (~9 out of 10) demonstrated accurate knowledge of the harms of tobacco smoke and smoking. This is a positive result and shows wide and accurate awareness of the harms of cigarettes among high school students, which could be the result of concerted efforts to prevent and reduce tobacco use among youth. For example, direct health promotion activities are underway in Virginia and multiple marketing campaigns reached 648,500 in 2009 with approximately 694,000 – 731,000 youth reached annually 2004 to 2008 [19]. However, among asthmatic students, less healthy responses to questions about the knowledge and beliefs about the harms of smoking (i.e., second-hand smoke is not harmful, smoking 1–5 cigarettes a day is not harmful, smoking only a year or 2 years is safe) were positively associated with increased odds of smoking in the past 30 days; however, the only belief that had a statistically significant association was thinking that smoking for 1–2 years is safe. This finding likely reflects the belief among youth that it is not hard to quit smoking. While a general message that smoking is harmful can be received by all high school students, particular knowledge that smoking even for a relatively short period of time (only 1 or 2 years) is not safe and perhaps information about the reality of early nicotine dependence could discourage smoking initiation or occasional smoking, which could lead to regular smoking. This supports previous findings that low risk perception is a predictor of smoking among adolescents [20].

Overall, to realize the largest public health impact, targeted prevention efforts that make the short- and long-term consequences of smoking concrete and highlight how to combat peer pressure are needed to address smoking among adolescents with and without asthma. Engaging, age-appropriate materials and messages delivered through public schools, recreation centers, and faith-based institutions can effectively impact attitudes and ultimately lead to behavior change. For example, since the inception of statewide programming and partnering with the Virginia Department of Education on health promotion activities, the prevalence of smoking among Virginia high school students dropped from 28.6 % in 2001 to 8.2 % in 2015 [19]. Previous research also suggests that gender should also be considered when developing prevention and cessation interventions for asthmatic adolescents [21].

### Health professional communication

To date, no studies have assessed patient-provider communication about smoking with asthmatic adolescents. In this study, high school students with asthma had more communication with health professionals regarding smoking than those without asthma. While this is a positive result, in total, less than half of all students with asthma were asked if they smoke. Using the same smoking measure, 2011 National Youth Tobacco Survey data suggest that among students ages 15 years and older, the proportion of health care providers asking about smoking ranged from 35.3 to 69.9 depending on the student’s smoking history [22]. Therefore, it appears that providers could continue to improve. Among asthmatic students, communication with a health professional regarding smoking had an inconsistent effect, as health professionals asking about smoking was significantly associated with increased odds of smoking, while advice about not smoking was not associated with smoking. The positive association between providers asking about smoking and behavior could be due to the fact that doctors are more apt to ask about smoking if they know the patient has a history of smoking and/or that smokers could be more likely to remember being asked. Previous research among adolescents from Tennessee found screening and advice about smoking from a healthcare provider had a positive impact on knowledge and quit attempts among smokers [23], which conflicts with the findings of this study. Given the high risk of smoking initiation during adolescence, smoking should continue to be addressed consistently by doctors and other providers treating adolescents with asthma. While laws exist to promote confidential discussions between medical providers and adolescent patients, one study found 64 % of adolescents seen in medical practice had documented alone time with a clinician [24] whereas another study reported almost 48 % of adolescents ages 15–17 years who had a preventive health visit did not have time alone with a clinician [25]. Further, about one-third of parents disagree that providers should see an adolescent patient alone [26]. Thus, if adolescents less than 18 years of age receive medical care with a parent present, discussing risky behaviors such as smoking may be sensitive and difficult to broach.

Then again, one of the most important aspects of asthma management is self-care, such as avoiding asthma triggers and complying with control medication. High school students are able to understand and remember self-care plans and they have enough independence to make decisions to avoid smoking. This study suggests that there are missed opportunities for health care professionals to communicate to adolescent patients with asthma the importance of being tobacco free and how it is essential in preventing acute episodes. In addition, some studies of smoking among asthmatic adolescents have found that poor medication adherence is associated with smoking [12]. Taken together, these findings could indicate that a complete asthma management plan provided by a health professional would help address smoking as well as other aspects of self-care. Further studies could examine the relationship between patient-provider communication and asthma episode severity among adolescents as well as confidence to avoid irritants such as tobacco smoke, and self-efficacy in asthma management among adolescents.

### Limitations

These study findings should be considered in light of a few limitations. First, a self-report questionnaire, administered in school classrooms, was used for this study so misclassification could exist. Students could have inaccurately reported their asthma status or diagnosis; however, this is unlikely given asthmatic students generally take medications and see a primary care provider at least once a year once they are diagnosed [27]. In addition, there could be a tendency toward socially desirable answers about smoking behavior by students. However, self-reported smoking by adolescents is similar to the prevalence given cotinine testing and is considered valid for population-level estimates [28–31]. Also, if present, under-reporting of smoking could mean there is an even greater public health impact than estimated in the current study. Importantly, the prevalence of current asthma and smoking in the last 30 days were assessed using the identical questions to that in the CDC’s Youth Tobacco Survey and Youth Risk Behavioral Surveillance (YRBS) survey, which reportedly have high reliability [31] and facilitate comparison to national data [3]. Smoking, defined as having ≥1 cigarette in the past 30 days, was purposely coded to align with the U.S. Healthy People 2020 tobacco-related Leading Health Indicator goal to reduce the prevalence of adolescents smoking in the last 30 days [32]. Second, because the high school students in the sample are from only one state in the U.S., findings may not be generalizable to all U.S. high school students. However, current cigarette smoking prevalence in this study was comparable to the identical contemporaneous measure from the national 2009 YRBS survey (19.2 %) [3]. The prevalence of current asthma in our sample was higher than national averages from the 2005–2010 National Health and Nutrition Examination Survey (12.9 %) [33], however, Virginia is well-known for its asthma burden within the U.S. [34]. Smoking prevalence among asthmatics in this study (17.9 %) was much lower than the prevalence of current smoking among asthmatic adolescents in the nationally representative longitudinal (48 %) [35]. Participating schools were in urban, suburban, and rural communities and schools that declined to participate were not more likely to be inner city schools or schools with higher proportion of historically disadvantaged students (M. White, Virginia Foundation for Healthy Youth, personal communication, 2014). Additionally, school-based studies exclude drop-outs and these youth tend to engage in more risky health-related behaviors. Third, the study data are cross-sectional; thus, temporality regarding asthma diagnosis and smoking as well as doctors asking about or advising against smoking is not exactly known. Lastly, because this study involves secondary data analyses, potential confounders (e.g., socio-economic status, school-level health promotion activities, etc.) and associations of interest were limited by a function of the initial design and data collection.

## Conclusions

While most high school students understand the harms of even occasional smoking, many of them still smoke, including students with asthma. In this cross-sectional study, among students with asthma, advice from health professionals does not appear to make a significant difference in the decision to smoke; however, importantly, personal beliefs and knowledge about the harms of smoking for only 1–2 years significantly increased the odds of smoking. Overall, this highlights the importance of school-based educational interventions among children and adolescents in the effort to prevent youth smoking. Both asthma and smoking remain a major health issue for adolescents in Virginia and in the U.S., and many young people are affected by both. In general, formal prevention education about the harms of smoking takes place mostly in the school [36], while patient education about asthma management takes place primarily in a clinical setting. Health education that currently focuses on either prevention or treatment/management should find an intersection, so that issues that affect the same population can be addressed. Helpful in this topic could be studies addressing provider-patient communication about smoking among adolescents with asthma and broader public health educational interventions targeting teens with asthma.

## Abbreviations

CDC: Centers for Disease Control and Prevention
CI: Confidence interval
OR: Odds ratio
